# Bioorganic Fertilizer Enhances Soil Suppressive Capacity against Bacterial Wilt of Tomato

**DOI:** 10.1371/journal.pone.0121304

**Published:** 2015-04-01

**Authors:** Lijuan Liu, Chengliang Sun, Shuangri Liu, Rushan Chai, Weiqing Huang, Xingxing Liu, Caixian Tang, Yongsong Zhang

**Affiliations:** 1 Ministry of Education Key Laboratory of Environmental Remediation and Ecosystem Health, College of Environmental and Resource Sciences, Zhejiang University, Hangzhou, China; 2 Zhejiang Provincial Key Laboratory of Subtropical Soil and Plant Nutrition, College of Environmental and Resource Sciences, Zhejiang University, Hangzhou, China; 3 Centre for AgriBioscience, La Trobe University, Melbourne Campus, Bundoora, Australia; Zhejiang University, CHINA

## Abstract

Tomato bacterial wilt caused by *Ralstonia solanacearum* is one of the most destructive soil-borne diseases. Many strategies have been taken to improve soil suppressiveness against this destructive disease, but limited success has been achieved. In this study, a novel bioorganic fertilizer revealed a higher suppressive ability against bacterial wilt compared with several soil management methods in the field over four growing seasons from March 2011 to July 2013. The application of the bioorganic fertilizer significantly (*P*<0.05) reduced disease incidence of tomato and increased fruit yields in four independent trials. The association among the level of disease incidence, soil physicochemical and biological properties was investigated. The soil treated with the bioorganic fertilizer increased soil pH value, electric conductivity, organic carbon, NH_4_^+^-N, NO_3_^-^-N and available K content, microbial activities and microbial biomass carbon content, which were positively related with soil suppressiveness. Bacterial and actinomycete populations assessed using classical plate counts were highest, whereas *R*. *solanacearum* and fungal populations were lowest in soil applied with the bioorganic fertilizer. Microbial community diversity and richness were assessed using denaturing gel gradient electrophoresis profile analysis. The soil treated with the bioorganic fertilizer exhibited higher bacterial community diversity but lower fungal community diversity. Redundancy analysis showed that bacterial community diversity and richness negatively related with bacterial wilt suppressiveness, while fungal community richness positively correlated with *R*. *solanacearum* population. We concluded that the alteration of soil physicochemical and biological properties in soil treated with the bioorganic fertilizer induced the soil suppressiveness against tomato bacterial wilt.

## Introduction

Bacterial wilt caused by the vascular pathogen, *Ralstonia solanacearum* has resulted in serious damage in agricultural crop production [1]. Disease incidence of bacterial wilt of tomato in southern China is increasing, and has caused severe yield losses. Various strategies have been taken to control bacterial wilt, such as grafting [2], biofumigation [3] and growing resistant crop varieties [4], but limited success has been achieved due to the high surviving capacity in complex environments [5], wide host range [1], and genetic diversity [6] of *R*. *solanacearum*. Although soil solarization has been recognized as an effective method to control bacterial wilt [7], a short-term effectiveness of solarization limits its application throughout the year. Therefore, effective measures to suppress bacterial wilt disease are needed to be developed.

Biocontrol and use of organic amendments such as compost, manure and plant residues emerge as environmentally friendly strategies and popular methods to suppress soil-borne disease. The use of organic amendments as a promising alternative to suppress the soil-borne disease less depends on chemicals. Mazzola [8] argued convincingly that organic amendments could increase microbial activity and enrich populations of specific microorganisms or microorganism groups to inhibit the invasion of pathogens. According to the bacterial community profiles after pig slurry application, Gorissen et al. [9] confirmed that the lower population size of *R*. *solanacearum* resulted from an alteration of microbial community structure induced by pig slurry. In recent decades, several biocontrol agents of *R*. *solanacearum* have been isolated from the rhizosphere soil or plant tissues, such as *Bacillus* spp. [10], *Streptomyces* spp. [11] and avirulent mutants of *R*. *solanacearum* [12]. However, the ability to competitively colonize and survive in the rhizosphere is still a prerequisite for antagonistic strains in suppressing soil-borne diseases [10]. Therefore, niches and nutrients become important when competition occurs between pathogens and antagonists. However, organic fertilizers can supply adequate energy and nutrients for antagonists to improve the suppressive capacity towards pathogens [13]. Importantly, organic fertilizers from municipalities, industries and farms meet the sustainable development of agriculture by recycling organic wastes and reducing the use of chemical fertilizers and fungicides in crop production [14]. The combination of organic fertilizers and antagonists (termed bioorganic fertilizer in our study), as a novel strategy to control bacterial wilt, showed best results in tobacco [10] and potato [15], and decreased bacterial disease incidences successfully. However, few studies focus on the suppressive capacity of soil treated with bioorganic fertilizers towards tomato bacterial wilt in the field.

Soil suppressive ability against pathogens may be affected by multiple factors, such as soil pH, organic matter content, nutrient availability and enzyme activity, which may in turn affect the interactions between pathogens and antagonists, and microbial resistance against invasion of pathogens in soil [16]. Therefore, analysis of association among the level of disease incidence, physicochemical and biological properties is useful to elaborate the mechanisms of soil suppressiveness of bioorganic fertilizers. The purpose of this study was to evaluate the soil suppressive capacity of a bioorganic fertilizer towards bacterial wilt in two tests over four growing seasons from March 2011 to July 2013. Our results demonstrated that 1) the application of bioorganic fertilizer more efficiently controlled bacterial wilt than other soil amendment methods, 2) the application of bioorganic fertilizer significantly improved soil physicochemical and biological properties, 3) the alteration in soil physicochemical and biological properties after the application of bioorganic fertilizer induced the suppressive capacity towards bacterial wilt.

## Materials and Methods

### Experimental site

The experiments were conducted in Xiaogang Town, Ningbo, Zhejiang province of China (121°44′ E, 29°56′ N) with a subtropical monsoon climate (no specific permissions were required for soil sampling in this location and the field in this study did not involve endangered or protected species). The average mean minimum and maximum temperatures are 25°C and 35°C, respectively, during the summer period from June to August. The site has 150 rainy days per year, and 1317 mm of mean annual precipitation. Tomato has been cultivated for over six years at this site, and bacterial wilt became a serious problem.

### Field experiments

The experimental design was a randomized block with four replicates. Sainto tomato (CTX 201) seedlings were grown in sterile nursery substrate for 25 d. Then, one hundred and twenty seedlings were transplanted into each plot (17.0 m × 1.3 m). Field experiments contained two different tests. Test 1 was conducted for two growing seasons: from March 2011 to July 2011 (Trial 1) and September 2011 to February 2012 (Trial 2). The treatments were shown as follows: (1) CK (NPK fertilizer), (2) O (NPK fertilizer + 3 t organic fertilizer ha^-1^), (3) B (NPK fertilizer + 3 t bioorganic fertilizer ha^-1^). After Test 1, a further test (Test 2) was conducted over two growing seasons from September 2012 to February 2013 (Trial 3) and from March 2013 to July 2013 (Trial 4), with the following treatments: (1) CK (NPK fertilizer), (2) S (NPK fertilizer + soil disinfection), (3) S + B (soil disinfection + NPK fertilizer + 3 t bioorganic fertilizer ha^-1^). The NPK fertilizers consist of chemical fertilizers at the N:P:K ratio of 15:7:12; N was applied as urea, P applied as superphosphate with 7% P and K as potassium sulfate containing 41% K. In order to balance the NPK level in all treatments, different amounts of NPK fertilizers were applied into each plot. The calcium cyanamide containing 17% N was used for soil disinfection at a dosage of 4 kg plot^-1^. Rape seed meal was applied as organic fertilizer and contained 26.4% organic C, 2.3% N, 1.3% P, 2.5% K and 25% H_2_O. Bioorganic fertilizer with 24.4% organic C, 3.1% N, 0.6% P, 2.7% K and 42% H_2_O was supplied by Jiangsu New Ground Bio-fertilizer Engineering Center Co., Ltd. in China. The amounts of N, P and K supplied were 0.60, 0.70 and 2.60 kg plot^-1^, respectively.

### Disease incidence

The incidence and severity of bacterial wilt were observed daily after transplanting tomato seedlings. The disease incidence was evaluated when the disease emerged and calculated as the percentage of diseased plants compared with the total number of growing plants in each plot. We presented the data of disease incidence at 30, 90 and 120 DAT in Trials 1 and 4, and 30, 100 and 150 DAT in Trials 2 and 3.

### Soil sampling

Soil samples (5–20 cm) adhering to the roots were taken from each plot in the period of florescence, full fruit and harvest at 30 days after transplanting (DAT), 90 DAT and 120 DAT in Trial 1 and Trial 2, respectively, and at 30 DAT, 100 DAT and 150 DAT in Trial 3 and Trial 4, respectively. The number of culturable microbial community populations in soil was determined immediately after soils were transported to the laboratory. A portion of soil samples were immediately kept at 4°C in 50-mL polypropylene tubes for determining the soil microbial activities and microbial biomass C within 48 h. Another portion of soil samples were kept at −80°C for PCR-DGGE analysis. The remaining soils were air-dried to determine soil physicochemical properties.

### Soil physicochemical properties analysis

Soil water content was determined gravimetrically after drying at 105°C for 8 h. Soil pH and electrical conductivity (EC value) were estimated on air-dried samples using a 1:5 soil/water ratio. Soil organic carbon (SOC) was measured by the Walkley-Black method [17], and total N was assayed by Kjeldahl analysis [18]. The NH_4_^+^-N was extracted with 2 M KCl and analyzed colorimetrically. Soil NO_3_^–^-N was extracted with 0.5 M K_2_SO_4_ before colorimetric measurement at 220 nm and 270 nm. Available P was determined using the methods outlined by Kuo [19]. Available K in the NH_4_OAc extracts [20] was estimated using an atomic absorption spectrophotometer.

### Soil microbial activity and microbial biomass analysis

The activities of phosphomonoesterase, arylsulfatase, β-D-glucosidase and dehydrogenase were measured according to Tabatabai [21]. The urease activity was determined as described by Tabatabai and Bremner [22]. Fluorescein diacetate (FDA) hydrolysis was assayed according to Adam [23]. Soil respiration was estimated by measuring CO_2_ evolved from the soil [24]. Microbial biomass carbon was estimated by the fumigation-extraction method [25].

### Microbial community populations in soil

The culturable microbes were enumerated by a standard 10-fold dilution method. Briefly, 10 g soil was added to 90 mL sterile distilled water and shaken on a rotary shaker at 200 rpm for 30 min. Soil suspensions at appropriate dilution rates were spread on plates and incubated on suitable media as follows: Beef extract-peptone medium for bacterial population, Martin’s Rose Bengal agar for fungal population, Gaoshi No. 1 agar for actinomycetes population [26] and semi-selective SMSA medium for *R*. *solanacearum* population [27]. The inoculated agar plates with bacteria and *R*. *solanacearum* were incubated at 28°C for 2 d in the dark, and with fungi at 28°C for 4 d, whereas plates with actinomycetes were incubated in the dark at 30°C for 5 d. Microbial community populations in soil were measured after crop harvest, and *R*. *solanacearum* recovery was tested at 30 DAT, 90 DAT and 120 DAT in Trials 1 and 4, and 30, 100 and 150 DAT in Trials 2 and 3 in the field.

### Analysis of microbial community diversity in soil by PCR-DGGE

The DNA was extracted from 0.5 g soil samples with the UltraClean Soil DNA Isolation Kit (MO BIO Laboratories, Carlsbad, CA) according to the manufacturer’s instructions.

Polymerase chain reaction (PCR) targeting the bacterial 16S rRNA used the primer pairs 338f-GC/518r [28]. A 50 μL PCR reaction mixture contained: 5 μL of 10 × PCR Buffer (TaKaRa), 0.3 μL of Tap polymerase (5 units μL^-1^, TaKaRa), 4 μL of dNTP Mixture (2.5 mM each), 1 μL of DNA template, 2 μL of primers and double-distilled water to a total of 50 μL. Touchdown PCR was conducted under the condition as followed. An initial denaturation step at 94°C for 5 min followed by a thermal cycling programme as follows: 1 min denaturation at 94°C, 1 min primer annealing at an initial 65°C, decreasing 1°C every cycle to a final of 55°C, 3 min primer extension at 72°C. Thirty cycles were run with a final extension step at 72°C for 7 min.

PCR amplifications of the fungi were performed using the ITSF [29] and ITS4 [30] primers, which is the fungal internal transcribed spacer (ITS) rRNA regions. Polymerase chain reaction mixture was the same as the bacterial PCR reaction mixture. Cycle conditions in the fungal PCR were as follows: 94°C for 5 min, then 35 cycles of 94°C for 1 min, 55°C for 1 min, and 72°C for 1 min, followed by a final extension step at 72°C for 5 min. The first round PCR amplifications were purified using the PCR Purification Kit (Omega, USA) according to the manufacturer’s instructions. The next PCR was performed to produce ITS1 products under the cycling conditions as follows: 94°C for 5 min, then 35 cycles of 94°C for 30 s, 55°C for 30 s, and 72°C for 30 s, followed by a final extension step at 72°C for 5 min.

DGGE analysis was performed using the Dcode Universal Mutation Detection System (Bio-Rad Laboratories, Hercules, USA). Thirty microliter of PCR product samples with 6 mL loading dye were loaded onto 8% (w/v) polyacrylamide gels (37.5:1 40% acrylamide/bis-solution) containing a gradient of 40–60% denaturants for the bacteria DNA and 30–60% for the fungi DNA. Electrophoresis of the bacteria DNA was performed in 1×TAE buffer at 60°C with a constant voltage of 100 V for 10 h, and then gels with fungal DNA were run at 60°C and 75 V for 16 h. After electrophoresis, the gels were stained with SYBR GREEN I (Sigma) and the DNA bands were observed with a Gel-Doc image analyzer (Bio-Rad Laboratories).

DGGE images were analyzed for band detection and intensity with Quantity One computer software (Version 4.6.3, Bio-Rad Laboratories). For bacteria and fungi, the band richness analysis was defined as the number of DGGE bands detected. The Shannon-Weaver index of microbial community diversity (*H*) characterizes the microbial community diversity in soil [31]. The Shannon-Weaver index was calculated as

$$H=-\sum P_{i}\text{ln}P_{i}=-\sum \left(n_{i}/N\right)\text{ln}\left(n_{i}/N\right)$$

where *P*_*i*_ was the ratio of the number in a specific group and the total number, *n*_*i*_ was the intensity of a band and *N* was the sum of all band intensities in the densitometry profile.

### Statistical analysis

All statistical analyses were performed with the SPSS 13.0 software program (SPSS Inc., Chicago, IL). Differences among the treatments were assessed with a one-way analysis of variance (ANOVA) at the end of each bioassay. A comparison of means was performed by a Fisher’s least significant difference test (LSD) and Duncan multiple range test with a significance level of *P*<0.05. Principal component analysis (PCA) and redundancy analysis (RDA) were performed by using Canoco 4.5. PCA was performed using a factor analysis model. The associations among soil physicochemical, biological, soil microbial community diversity, *R*. *solanacearum* population and disease incidence were performed by RDA.

## Results

### Control efficiency of bacterial wilt disease

**Fig 1:**
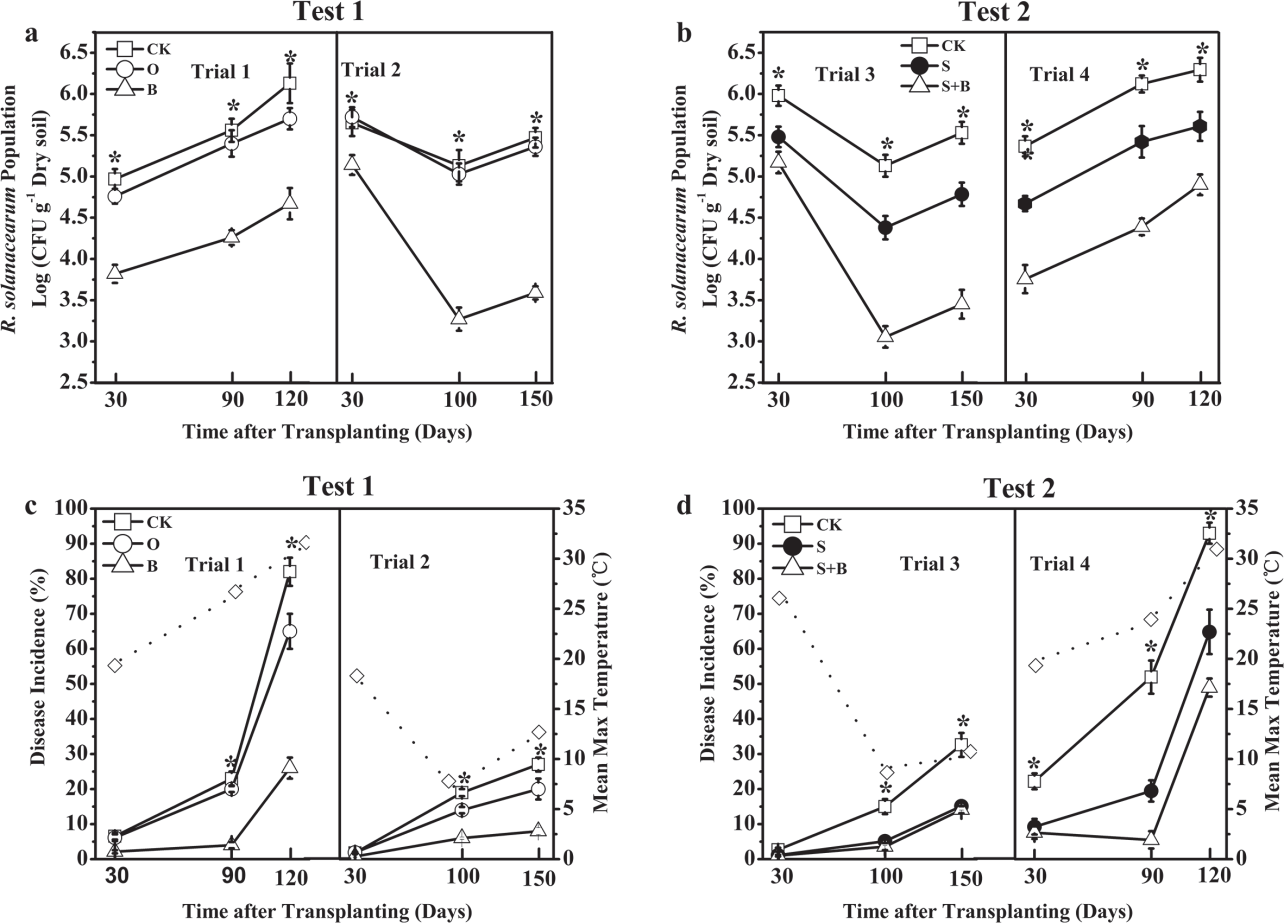
Population of *Ralstonia solanacearum* in soil and disease incidence over time in Test 1 (a and c) and Test 2 (b and d). The dotted lines and ◇ represent the mean max temperature in the above time periods in different trials. CK: NPK fertilizer; O: NPK fertilizer + organic fertilizer; B: NPK fertilizer + bioorganic fertilizer; S: NPK fertilizer + soil disinfection; S+B: soil disinfection + NPK fertilizer + bioorganic fertilizer. * indicates the significant difference between the treatments at the 0.05 probability level according to the Duncan’s test.

The controlling efficacy of tomato bacterial wilt by different fertilizers/amendments at different growth stages was shown in Fig 1. *R*. *solanacearum* population in the B treatment was decreased by 23–24% in Trial 1 and by 8–36% in Trial 2 during the entire experiment period compared with the control (Fig 1A), while no difference was observed between the O and control treatments in both trial periods.

The S+B treatment resulted in the lowest *R*. *solanacearum* population, which was only 86, 59 and 62% of the control at 30, 100 and 150 DAT, respectively, in Trial 3 (Fig 1B). The S application decreased *R*. *solanacearum* population by 8, 15 and 14%, respectively. In Trial 4, the treatment effect on *R*. *solanacearum* population displayed the same pattern as observed in Trial 3.

**Fig 2:**
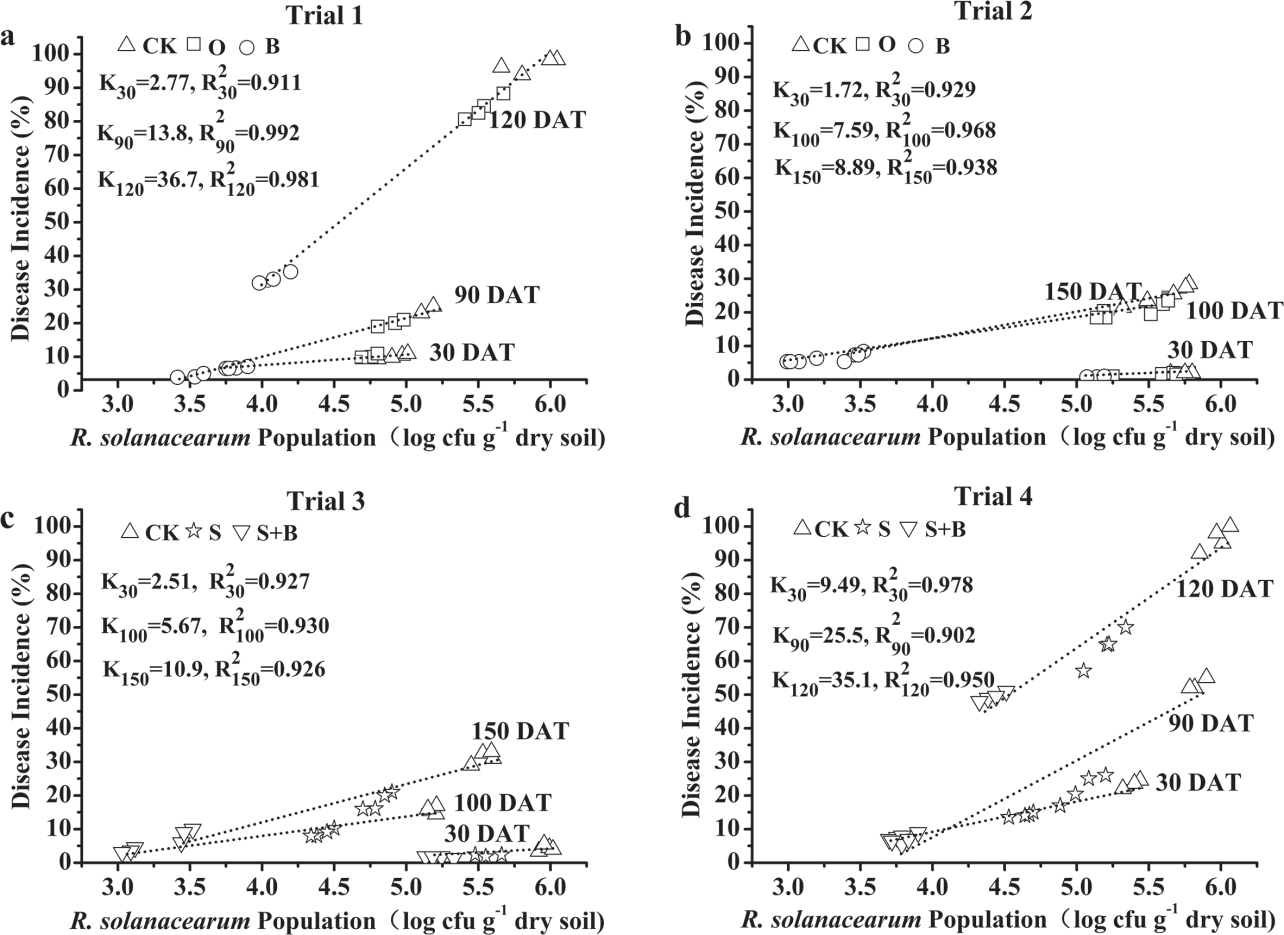
Relationships between population of *R*. *solanacearum* and disease incidence in Trial 1 (a), Trial 2 (b), Trial 3 (c) and Trial 4 (d). CK: NPK fertilizer; O: NPK fertilizer + organic fertilizer; B: NPK fertilizer + bioorganic fertilizer; S: NPK fertilizer + soil disinfection; S+B: soil disinfection + NPK fertilizer + bioorganic fertilizer. DAT, days after transplanting; K, the slope of line from the relationship between *R*. *solanacearum* population and tomato disease incidence; R^2^, related coefficient for the line from the relationship between *R*. *solanacearum* population and tomato disease incidence.

The effects of different treatments on disease incidence were shown in Fig 1C and 1D. The disease incidence in the B treatment was decreased by 67–82% and 55–70%, respectively in Trial 1 and Trial 2 compared with the control (Fig 1C). The treatment effect on disease incidence in Trials 3 and 4 displayed the same pattern as that observed in Trials 2 and 1, respectively (Fig 1D). Interestingly, the disease incidence increased rapidly over the growth stages and exhibited high values in Trials 1 and 4, while it increased slowly and showed lower values in Trials 2 and 3 (Figs 1C and 1D and 2). The difference in disease incidence was related to the temperature variation; high disease incidence was related with high temperatures (Fig 1C and 1D). There were positive relationships between *R*. *solanacearum* population and disease incidence with coefficients (R^2^) over 0.90 in all four trials (Fig 2).

**Fig 3:**
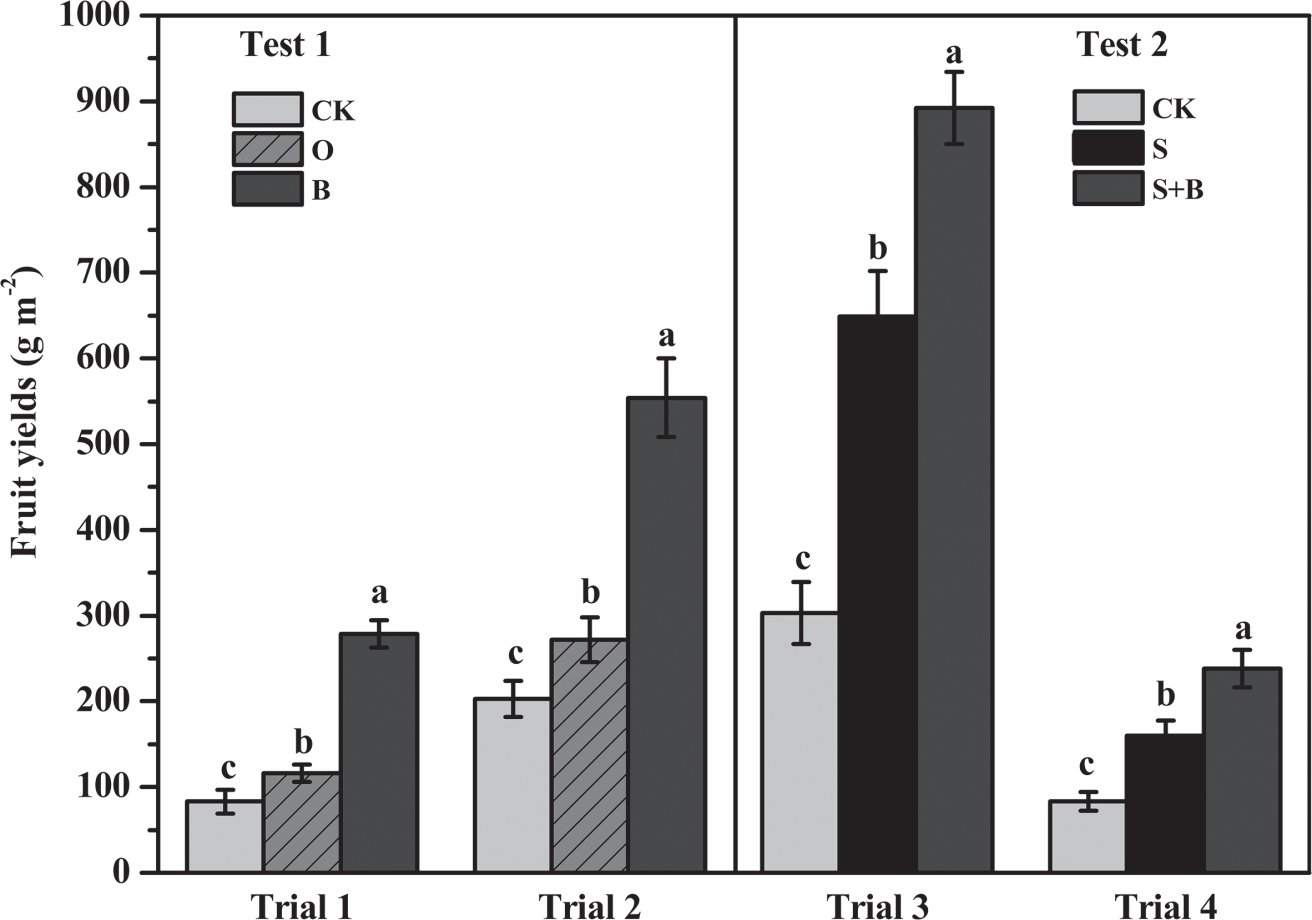
Effect of different treatments on tomato yields. CK: NPK fertilizer; O: NPK fertilizer + organic fertilizer; B: NPK fertilizer + bioorganic fertilizer; S: NPK fertilizer + soil disinfection; S+B: soil disinfection + NPK fertilizer + bioorganic fertilizer. Bars with different letters indicate a significant difference between the treatments, as defined by Duncan’s test (*P*<0.05).

Tomato yields increased by 233% and 173% for the B treatment, and by 39% and 34% for the O treatment compared with the control in Trials 1 and 2, respectively. The yield increased by 194% and 186% in the S+B treatment, by113% and 92% in the S treatment compared with the control in Trials 3 and 4, respectively (Fig 3).

### Soil physicochemical properties

Soils collected from different treatments in Test 1 and Test 2 differed substantially in soil physicochemical property values (Table 1). The application of bioorganic fertilizer significantly increased soil pH value, EC value, NH_4_^+^-N, NO_3_^–^-N, available K and SOC contents compared with the O treatment and control in Trials 1 and 2. Organic fertilizer alone only increased NO_3_^–^-N, available K, and SOC content compared with the control.

The application of bioorganic fertilizer (S+B) resulted in the highest values of pH value, EC value, available K, NO_3_^–^-N, SOC and total N, while the S treatments had higher values of these properties than the control except available K and SOC content (Table 1) in Trials 3 and 4.

**Table 1:** The soil physicochemical properties, and microbial activities and biomass carbon in different treatments.

			pH (1:5 water)	EC (1:5 water) (μS cm^-1^)	NH_4_^+^-N (μg g^-1^)	NO_3_^–^-N (μg g^-1^)	Total N (mg g^-1^)	Available P (μg g^-1^)	Available K (μg g^-1^)	Organic C (mg g^-1^)	Phosph (μg g^-1^)	β-gluco (μg g^-1^)	Aryl (μg g^-1^)	Urea (μg g^-1^)	Dehydr (μg g^-1^)	FDA (μg g^-1^)	SR (CO_2_ μL g^-1^ d^-1^)	MBC (μg g^-1^)
Test 1	Trial 1	CK	5.47±0.05b	543±28b	9.3±0.4c	321±31c	2.24±0.18ab	96±10ab	75±6c	26.6±2.3c	332±24c	123±24b	z40±4c	173±13c	2.43±0.31c	342±13c	36.4±3.6d	186±6d
		O	5.62±0.09b	554±13b	10.3±0.5b	413±20b	2.52±0.15a	120±15a	95±7ab	31.4±1.0ab	391±24ab	145±16b	62±5b	237±19ab	3.31±0.35b	348±27c	52.2±6.4c	253±18c
		B	5.92±0.07a	712±15a	12.1±0.2a	473±20a	2.75±0.29a	130±10a	113±9a	33.1±1.1a	420±18a	198±20a	91±4a	286±22a	4.10±0.22a	446±24a	91.7±5.9a	335±14a
	Trial 2	CK	5.54±0.04d	834±20c	8.6±0.4b	446±26c	1.58±0.06c	522±21c	420±21c	31.2±1.6c	262±11c	254±15c	108±5c	186±14c	3.34±0.32d	262±9c	29.2±4.3c	145±12c
		O	5.80±0.06c	843±32c	9.3±0.3b	529±13ab	1.83±0.12b	611±19b	475±28b	41.0±0.7b	318±13b	290±12ab	150±14b	241±18ab	4.03±0.24c	326±20b	41.7±5.8b	224±20b
		B	6.40±0.07a	1048±38a	10.4±0.4a	562±18a	2.15±0.04a	729±30a	602±30a	42.8±1.0a	374±13a	340±21a	250±21a	290±18a	5.67±0.22a	395±17a	73.9±4.6a	313±12a
Test 2	Trial 3	CK	5.52±0.06c	655±14b	9.7±1.1b	207±11c	3.72±0.17c	723±19b	543±14b	41.3±1.6c	233±30b	127±19b	24±2c	182±14b	2.76±0.40c	93±8b	19.6±3.1c	79±11c
		S	5.99±0.12b	640±30b	13.4±1.0a	254±15b	4.19±0.12b	809±20a	561±13b	44.4±1.1b	250±14b	134±21b	31±2b	180±12b	3.84±0.28b	87±8b	26.3±2.0b	124±10b
		S+B	6.24±0.04a	900±25a	13.7±0.5a	332±12a	4.59±0.15a	816±20a	613±14a	53.6±2.4a	295±15a	178±16a	53±4a	231±14a	5.77±0.21a	169±17a	62.6±6.3a	248±15a
	Trial 4	CK	5.72±0.13c	476±13c	10.1±0.3b	119±17c	3.48±0.39b	631±17b	440±17b	38.8±2.9c	303±20b	153±17b	48±5b	112±4c	2.51±0.32c	232±18b	27.6±4.7c	135±5c
		S	6.12±0.09b	550±23b	13.4±1.7a	169±10b	3.87±0.45b	743±20a	457±20b	42.8±3.9b	301±14b	162±9b	54±9b	140±8b	4.15±0.41b	240±12b	42.5±6.4b	181±5b
		S+B	6.40±0.12a	735±32a	13.7±0.8a	203±11a	4.66±0.10a	754±37a	734±30a	49.4±1.5a	345±12a	248±13a	112±6a	196±10a	6.09±0.40a	397±19a	71.8±5.3a	352±18a

Data were expressed as mean ± standard error (n = 4). The data within a column of each trial with a same letter did not differ significantly at Fisher’s least significant difference test (LSD) and Duncan’s significance level 0.05. Phosph, phosphomonoesterase activity; Aryl, arylsulfatase activity; β-gluco, β-D-glucosidase activity; Dehydr, dehydrogenase activity; Urea, urease activity; FDA, fluorescein diacetate hydrolase activity; SR, soil respiration; MBC, microbial biomass carbon. CK: NPK fertilizer; O: NPK fertilizer + organic fertilizer; B: NPK fertilizer + bioorganic fertilizer; S: NPK fertilizer + soil disinfection; S+B: soil disinfection + NPK fertilizer + bioorganic fertilizer.

### Soil microbial activities and biomass carbon

Microbial activity and biomass carbon were chosen as the indicators to assess the soil suppressiveness, as they rapidly respond to the changes of soil environment. Application of bioorganic fertilizer significantly enhanced the activity of phosphomonoesterase, β-D-glucosidase, arylsulfatase, urease and dehydrogenase as well as FDA hydrolysis and soil respiration by 27, 61, 125, 66, 69, 30 and 152%, respectively, in Trial 1, and by 43, 34, 133, 57, 70, 51 and 153%, respectively, in Trial 2 (Table 1). However, the application of organic fertilizer (O) only slightly increased the microbial activity compared with the control. On the other hand, the application of bioorganic fertilizer increased microbial biomass C by 80% and 117% in Trials 1 and 2, respectively.

The S+B treatment exhibited the highest enzyme activities. It significantly increased the activity of phosphomonoesterase, β-D-glucosidase, arylsulfatase, urease and dehydrogenase, FDA hydrolysis and soil respiration by 27, 40, 120, 27, 109, 82 and 220%, respectively, in Trial 3, and 14, 61, 133, 75, 143, 71 and 160%, respectively, in Trial 4 (Table 1). The S application only increased the activity of dehydrogenase but not other enzymes compared with the control. However, the S application increased soil respiration by 34% and 54% in Trials 3 and 4, respectively. Furthermore, the S+B application led to the highest microbial biomass carbon (248 and 352 μg g^-1^ in Trials 3 and 4, respectively), followed by S (124 and 181μg g^-1^, respectively) and the control (79 and 135μg g^-1^, respectively).

### Culturable microbial community populations in soil

**Fig 4:**
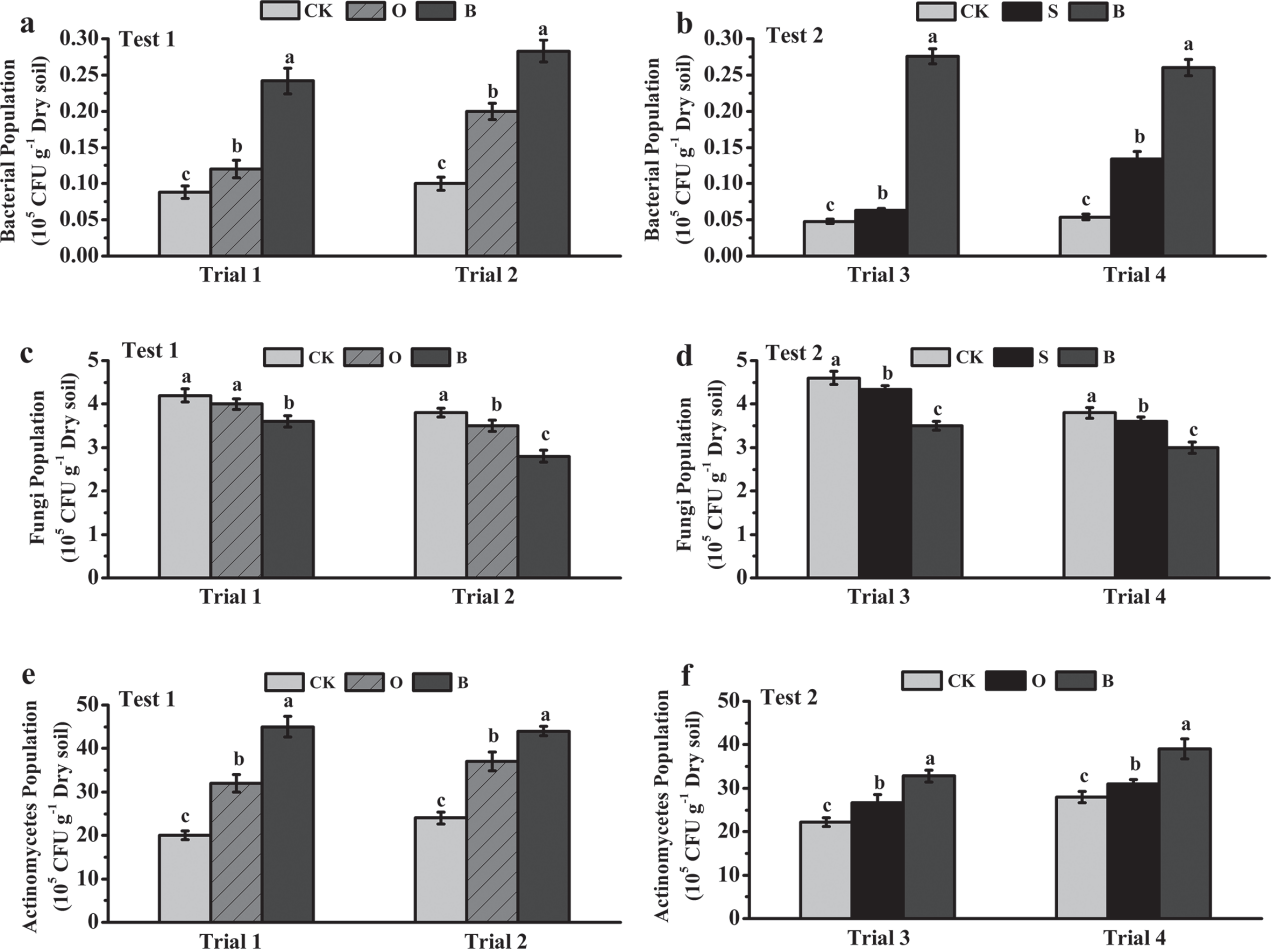
The population of bacteria (a and b), fungi (c and d), and actinomycetes (e and f) in soils from different treatments in Test 1 (a, c and e) and Test 2 (b, d and f). CK: NPK fertilizer; O: NPK fertilizer + organic fertilizer; B: NPK fertilizer + bioorganic fertilizer; S: NPK fertilizer + soil disinfection; S+B: soil disinfection + NPK fertilizer + bioorganic fertilizer. Bars with different letters indicate a significant difference between the treatments, as defined by Duncan’s test (*P*<0.05).

The effects of different fertilizers/amendments on soil microbial community populations were shown in Fig 4. The application of bioorganic fertilizer significantly increased the bacterial and actinomycetes populations, but decreased the fungal population. The bacterial community and actinomycete populations in soil were about 2.5- and 2.0-fold higher in the B treatment than the control in Trials 1 and 2, respectively (Fig 4A and 4E). However, the fungal population was significantly lower in the B treatment than in the O and control treatments (Fig 4C).

The effects of S and S+B treatments on bacterial, actinomycete and fungal populations in Trial 3 and 4 exhibited the same pattern as those of B treatment in 1 and Trial 2 (Fig 4B–4D and 4F).

### Microbial community diversity in soil

The DGGE gel profiles obtained with bacterial 16S rRNA and fungal ITS rRNA gene under different treatments were used to analyze microbial community diversity (S1 and S2 Figs). The analysis of Shannon-Weaver diversity and richness showed that the application of bioorganic fertilizer increased bacterial community diversity but decreased fungal community diversity in soil (Table 2). The application of bioorganic fertilizer led to higher values of Shannon-Weaver diversity and richness in bacterial community than the control and O treatments in Trials 1 and 2. The highest Shannon diversity and richness values of soil bacteria were observed in the S+B treatment, followed by the S treatment and lowest in the control (Table 2). Conversely, Shannon-Weaver diversity and richness of soil fungi community were lowest after bioorganic fertilizer application.

**Table 2:** The richness and Shannon-Weaver index (*H*) diversity of microbial community under different treatments.

			Bacterial		Fungi	
			Richness	*H*	Richness	*H*
Test 1	Trial 1	CK	44±2c	3.77±0.04c	49±2a	3.82±0.04a
		O	53±1b	3.96±0.02b	49±1a	3.82±0.02a
		B	66±2a	4.17±0.03a	41±2b	3.62±0.03b
	Trial 2	CK	38±1b	3.63±0.02b	51±2a	3.86±0.05a
		O	37±2b	3.60±0.03b	53±1a	3.87±0.03a
		B	48±2a	3.88±0.03a	42±3b	3.66±0.07b
Test 2	Trial 3	CK	23±2c	2.69±0.05c	50±2a	3.56±0.03a
		S	34±2b	3.20±0.05b	43±1b	3.36±0.03b
		S+B	44±1a	3.46±0.03a	38±2c	3.24±0.04c
	Trial 4	CK	35±1b	3.53±0.04b	39±2a	3.53±0.05a
		S	35±2b	3.53±0.05b	39±3a	3.55±0.07a
		S+B	57±3a	3.99±0.05a	23±2b	2.99±0.05b

Data were expressed as mean ± standard error (n = 4). The data within a column of each trial with a same letter did not differ significantly at Fisher’s least significant difference test (LSD) and Duncan’s significance level 0.05. CK: NPK fertilizer; O: NPK fertilizer + organic fertilizer; B: NPK fertilizer + bioorganic fertilizer; S: NPK fertilizer + soil disinfection; S+B: soil disinfection + NPK fertilizer + bioorganic fertilizer.

### Association among *R*. *solanacearum* population, disease incidence and soil biochemical properties

**Fig 5:**
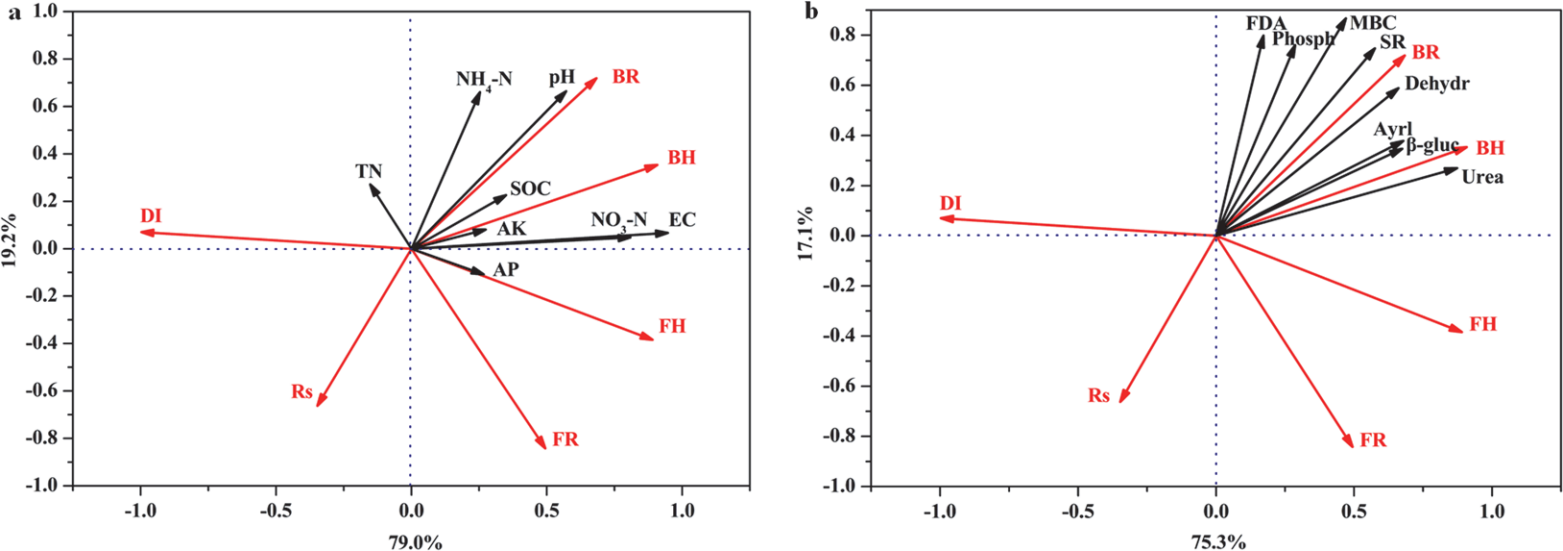
Association among level of bacterial wilt disease, microbial community diversity, soil physicochemical (a) and biological (b) properties. Monte Carlo permutation tests carried out for all canonical axes confirmed the significance of the relationship between the two datasets (a: F = 4.07, *P* = 0.03; b: F = 8.67, *P* = 0.04). TN, total N; AP, available P; AK, available K; DI, tomato disease incidence; Rs, *R*. *solanacearum* population; BR, bacterial population richness; BH, microbial diversity of bacterial community; FR, fungal population richness; FH, microbial diversity of fungal community; Phosph, phosphomonoesterase activity; Aryl, arylsulfatase activity; β-gluco, β-D-glucosidase activity; Dehydro, dehydrogenase activity; Urea, urease activity; FDA, fluorescein diacetate hydrolase activity; MBC, microbial biomass carbon; SR, soil respiration.

Associations of microbial diversity, *R*. *solanacearum* population and disease incidence with soil biochemical and with biological properties explained 98.2% and 92.4% variability of datasets, respectively (Fig 5A and 5B). There was a positive relationship between *R*. *solanacearum* population and disease incidence. Whereas bacterial community diversity was negatively related to the *R*. *solanacearum* population and disease incidence, *R*. *solanacearum* population correlated positively with the richness and diversity of fungal community. Soil pH value, EC value, SOC, NH_4_^+^-N, NO_3_^—^N and available K correlated positively but *R*. *solanacearum* population and disease incidence correlated negatively with bacterial community diversity. A close association also existed among soil microbial activities, microbial biomass and bacterial community diversity.

## Discussion

This present study examined the suppressive capacity of a bioorganic fertilizer on tomato bacterial wilt compared with other soil management methods across four seasons under field conditions. Our results demonstrated that the application of bioorganic fertilizer as a novel strategy provided the greatest ability to control bacterial wilt caused by *R*. *solanacearum* in tomato. The application of bioorganic fertilizer (B and B+S treatments) altered the microbial community structure, significantly decreased the population of *R*. *solanacearum* and showed the lowest disease incidence, which were consistent with the effect of bioorganic fertilizer on the suppressiveness against bacterial wilt of potato [15] and tobacco [10]. Furthermore, it was noted that there was a close association among disease incidence, *R*. *solanacearum* population and biochemical properties (Fig 5). These findings suggest that the enhancement of soil suppressiveness against bacterial wilt by the bioorganic fertilizer may be induced by multiple aspects, particularly the alteration of soil physicochemical and biological properties.

The application of bioorganic fertilizer improved soil physicochemical properties in all four trials (Table 1). The redundancy analysis (RDA) showed a close relationship between physicochemical properties and soil suppressiveness (Fig 5A). Previous studies showed that soil pH value [32], organic matter [33], NO_3_^–^-N and NH_4_^+^-N content [16] were negatively correlated with disease incidence. Michel and Mew [16] believed that ammonium toxicity of *R*. *solanacearum* was one mechanism of suppression. In addition, the application of organic amendments, which are rich in nitrogen, may reduce soil-borne diseases by allelochemicals generated during their storage or by subsequent microbial decomposition [34]. Furthermore, our results showed that soil pH value, EC value, SOC, NH_4_^+^-N, NO_3_^—^N and available K contents correlated positively with bacterial community diversity, which was negatively associated with *R*. *solanacearum* population and disease incidence. We suggest that the changes in soil properties by the bioorganic fertilizer strongly affect the interactions between pathogens and antagonistic populations, and contribute to the establishment of beneficial microbial community population, and subsequently influence the soil suppressiveness.

The application of bioorganic fertilizer alone or together with other amendments methods (B and S+B) significantly increased soil microbial activities (soil enzymes, FDA hydrolysis and soil respiration) and microbial biomass carbon (Table 1). Recently, Yin et al. [35] indicated that the severity level of upland rice seedling wilt negatively correlated with microbial biomass carbon and microbial activities in 19 soils from nine counties under various treatments. Conversely, Knudsen et al. [36] found no correlation between microbial activity and disease suppression of brown foot rot in soils from different sites under organic, integrated and conventional management. However, our present study clearly showed that microbial activities and microbial biomass were positively related with bacterial richness and diversity (*H*) (Shannon-Weaver index), but negatively with *R*. *solanacearum* population and tomato disease incidence (Fig 5B). Such a discrepancy between the studies could be due to the specific effect of different soil amendments on pathogens [37] or a stimulation of antagonists [38]. Moreover, a large biomass could create a competitive environment against the viability of pathogens in soils by organic amendments [39]. Increased microbial biomass in soil amended with compost was negatively associated with disease incidence or severity of *Pythium* root-rot in bulbous *Iris* [39]. We suggest that the decreased disease incidence was related to the poor competitive ability of the pathogens with native microorganisms in the soil amended with the bioorganic fertilizer.

The application of bioorganic fertilizer also altered microbial community structure as revealed by the plate count method (Fig 4) and PCR-DGGE profiles analysis (Table 2). Increased populations of bacteria and actinomycetes in soil are important to suppress soil-borne disease [40], whereas decrease of fungal community population might be beneficial for controlling soil disease, because most soil-borne crop diseases were caused by fungi, such as wilts, root rot, clubrot, and blight [41]. In addition, soil microbial diversity plays a key role in controlling bacterial pathogens [42]. Fargione and Tilman [43] reported that competition for nutrients with dominant microorganisms may limit invasion of pathogens in highly diverse communities. In the bioorganic fertilizer treatment (B and S+B), the diversity of bacterial community increased, but the fungal community decreased (Table 2), indicating that alteration of microbial diversity in soil with bioorganic fertilizer application promoted soil defensive capability against invasion of *R*. *solanacearum*. Our study further found that *R*. *solanacearum* population and disease incidence negatively related to the richness and diversity of bacterial community, but positively with fungal community richness (Fig 5). These results suggest that there was a close association between the improvement of soil suppressiveness and enhancement of microbial diversity in the bioorganic fertilizer treatment.

In our study, the application of bioorganic fertilizer significantly decreased population of *R*. *solanacearum* and thus inhibited the invasion of plant roots by the pathogens (Fig 1A and 1B). These results are consistent with the findings of Yuan et al. [10], showing a significantly lower population of *R*. *solanacearum* in the soils amended with bioorganic fertilizer compared to the control. The mechanism by which bioorganic fertilizer controlled tomato bacterial wilt is mainly through an antagonism by the antagonistic bacteria together with organic fertilizer, as a component of bioorganic fertilizer, suppressing *R*. *solanacearum* in the rhizosphere of tomato plants. In addition, the organic fertilizer provided antagonistic strains with additional carbon and nutrients to survive and hence to suppress *R*. *solanacearum* for a longer period of time (Fig 1A and 1B). Furthermore, where the bacterial wilt disease could be controlled efficiently, *R*. *solanacearum* populations were below 6.0 log cfu g^-1^ dry soil (Fig 2). Therefore, decreased *R*. *solanacearum* population in the soil is important for soil suppressiveness against the bacterial wilt.

The efficiency of the bioorganic fertilizer controlling tomato bacterial wilt was not consistent across the four trials, which may be resulted from different climatic conditions. The greater disease incidence in Trials 1 and 4 than Trials 2 and 3 (Figs 1C and 1D and 2) could be explained by unique epidemiology of *R*. *solanacearum* under different temperature regimes. Some studies reported that temperature fluctuation affected the viability of *R*. *solanacearum* [44]. Guo et al. [45] indicated that the bacterial wilt developed immediately and biocontrol efficiency decreased when the temperature was greater than 30°C. Other environmental factors, such as moisture may also cause the inconsistent controlling efficiency of biocontrol agents due to a lack of display of biocontrol traits (e.g., production of siderophores and antibiotics), which are regulated by genetics as well as many environmental factors [46]. We suggested that appropriate farming time with low temperature, low humidity and good ventilation should be considered to ensure the beneficial environmental conditions for tomato growth.

It was also noted that soil-borne pathogens could not be effectively controlled when a single management strategy was used, e.g. in the management of *Fusarium* wilt in banana [47]. Therefore, bioorganic fertilizer integrated with other agricultural measures may further decrease *R*. *solanacearum* population in soil and efficiently control the bacterial wilt. These agricultural measures include soil solarization [48], crop rotation [49], use of other amendments such as lime and calcium cyanamide with deep ploughing [50].

## Conclusions

The use of bioorganic fertilizers is a promising alternative strategy to controlling bacterial wilt caused by *R*. *solanacearum*. An alteration in soil physicochemical and biological properties caused by the bioorganic fertilizer contributes to soil suppressiveness towards bacterial wilt. It is worth noting that the application of bioorganic fertilizer in appropriate farming conditions, such as low temperature, low humidity and good ventilation and integration with other agricultural methods should be considered to effectively suppress *R*. *solanacearum* survival. However, in our study, the specific microorganisms responsible for suppressing bacterial wilt in diverse soil microflora after bioorganic fertilizer application are unknown. Further research is needed to detect the specific microorganisms and to study their functions in improving soil suppressiveness.

## Supporting Information

S1 Fig Original denaturing gradient gel electrophoresis profiles of the bacterial community in soil under different treatments. (TIF)

S2 Fig Original denaturing gradient gel electrophoresis profiles of the fungal community in soil under different treatments. (TIF)
