# Supplementary figures and images for: Bioorganic Fertilizer Enhances Soil Suppressive Capacity against Bacterial Wilt of Tomato
Source: PLoS One. 2015 Apr 1;10(4):e0121304. doi: 10.1371/journal.pone.0121304 (PMC4382293; doi:10.1371/journal.pone.0121304)

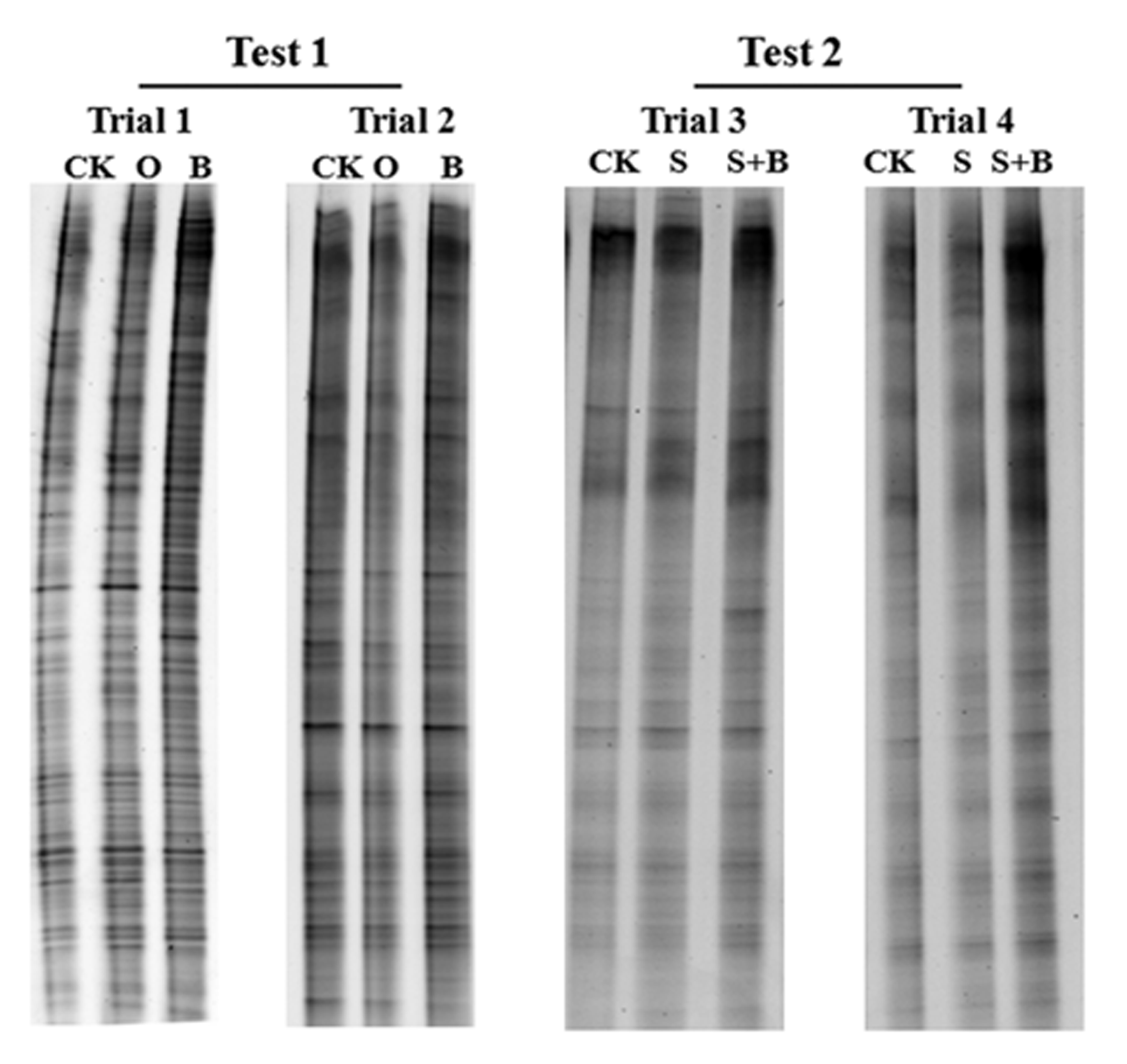

Supplement: S1 Fig — (TIF) [file pone.0121304.s001.tif]

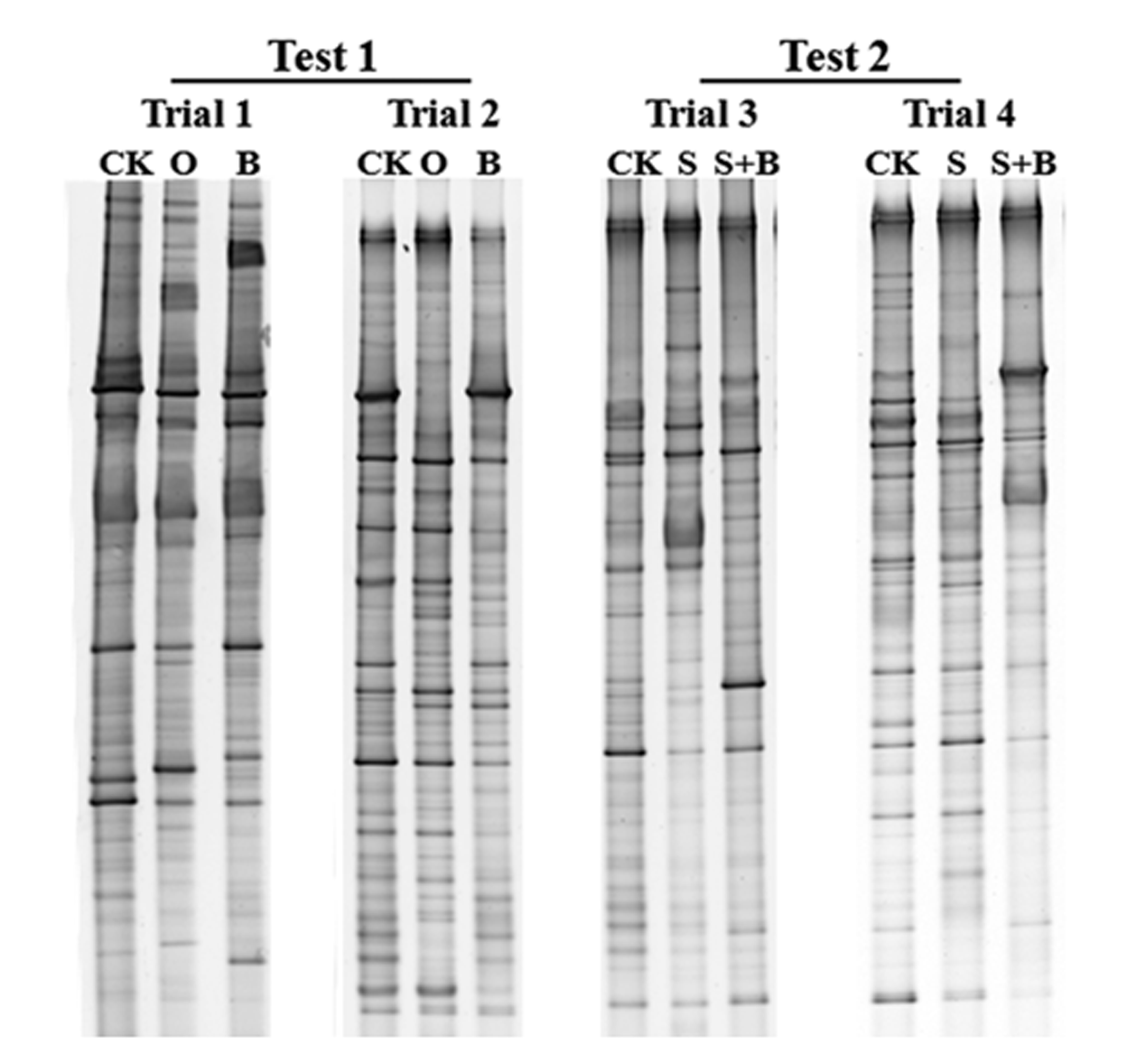

Supplement: S2 Fig — (TIF) [file pone.0121304.s002.tif]
